# Ignition Vulnerabilities of Combustibles around Houses to Firebrand Showers: Further Comparison of Experiments

**DOI:** 10.3390/su13042136

**Published:** 2021

**Authors:** Sayaka Suzuki, Samuel L. Manzello

**Affiliations:** 1 National Research Institute of Fire and Disaster (NRFID), Chofu, Tokyo 182-0012, Japan; 2 National Institute of Standards and Technology (NIST), Gaithersburg, MD 20899, USA

**Keywords:** WUI fires, structure ignition, home vulnerablities

## Abstract

Wildland fires and wildland urban-interface (WUI) fires have become a significant problem in recent years. The mechanisms of home ignition in WUI fires are direct flame contact, thermal radiation, and firebrand attack. Out of these three fire spread factors, firebrands are considered to be a main driving force for rapid fire spread as firebrands can fly far from the fire front and ignite structures. The limited experimental data on firebrand showers limits the ability to design the next generation of communities to resist WUI fires to these types of exposures. The objective of this paper is to summarize, compare, and reconsider the results from previous experiments, to provide new data and insights to prevent home losses from firebrands in WUI fires. Comparison of different combustible materials around homes revealed that wood decking assemblies may be ignited within similar time to mulch under certain conditions.

## 1. Introduction

Wildland fires and wildland urban-interface (WUI) fires have become a significant problem in recent years [1-5]. It is important to distinguish WUI fires from wildland fires; WUI fires include the combustion of both vegetative and human-made fuels and occur where large population centers exist, whereas wildland fires include the combustion of vegetative fuels and occur in uninhabited areas [4]. California in the US has lost thousands of structures to WUI fires over several decades [5]. To provide context to the scope of the WUI fire problem in the USA, seven of the fifteen largest fire loss events in the USA were WUI fires. These statistics do not even include the recent WUI fires in California during 2017 or 2018. Estimates place at least 70,000 communities or nearly 46 million structures at risk from WUI fires, which amounts to nearly 120 million people in the USA [5]. From 2019 to 2020, Australia has also suffered multiple, month-long bushfires, which resulted in the loss of thousands of structures WUI [6].

Every fire season, houses at the WUI are at risk, and people are forced to be prepared for it. One possible homeowner defense action is to clear combustibles around houses. The concept of defensible space or Home Ignition Zone (HIZ) was developed some time ago [7,8]. It suggests clearing all combustibles within a certain distance around a given home. While an interesting concept, it is difficult or impractical to implement easily due to the natural closeness of neighboring houses in WUI areas [4,9]. The other problem is, that while it may be possible there are no houses or trees within a specific homeowner’s HIZ, it is practically impossible to ensure that there are no combustibles around a given house. Many houses have external wood decks, gardening mulch and wood shingles or shakes for siding or roofing. Once those are ignited, adjunct houses are in danger too. If there are any fire-resistant materials placed within the HIZ, regular maintenance is also required to keep them fire-resistant [10].

The mechanisms of home ignition in WUI fires are direct flame contact, thermal radiation and firebrand attack. Out of these three fire spread factors, firebrands are considered to be a main driving force for rapid fire spread as firebrands can fly far from the fire front and start new fires [9,11]. Those are called short-distance spotting (up to 500 m–750 m), medium-distance spotting (1000–5000 m) and long-distance spotting (longer than 5000 m) [12]. For decades most prior firebrand research has focused on the transport of firebrands [13-20]. Recently, research has focused on firebrand ignition of structures, mainly homes, and one important research activity has used firebrand generators to accomplish this [21-31]. Firebrand generators have revealed the vulnerability of structures to firebrands by being able to experimentally recreate controlled firebrand showers, as seen in WUI fires [25]. Research on vulnerabilities to firebrand showers has been undertaken on wood decking assemblies, fencing assemblies, and ornamental mulch [26-31].

For decking assemblies, standard test methods exist. The Office of the State Fire Marshal (OFSM) in California has adopted the test method known as State Fire Marshal (SFM) STANDARD 12-7A-4 [32]. The SFM test method is intended to determine the response of decks to firebrand exposure and is similar to ASTM E2726 [33] and was proposed at a time when WUI fire science was in its infancy. Both those test methods are extensions of the ASTM E108 roof test [34]. A firebrand is simulated by placing a burning wood crib on top of a section of a decking assembly under an air flow. The dynamic process of multiple firebrands bombarding decking materials as a function of time is not adequately represented or simulated in this standard. Based on firebrand attacks from real WUI fires, it is expected that multiple firebrands would deposit within gaps/crevices between or on deck boards. In addition to not simulating a dynamic firebrand attack, the California and ASTM test methods do not fully consider possible differences between the size and mass of firebrands produced from burning vegetation and structures. As WUI fire science has developed further, the limitations of this test method have received increased attention. Even though mulch beds and fencing assemblies are important features near homes, there exist no standard test methods to rate their behavior in WUI settings.

Research on decking assemblies, fencing assemblies, and ornamental mulches have been conducted with various fire sources. In experiments with decking boards by Macindoe [35], the ignition source was not actual firebrands but rather methenamine tablets. The board spacing used in all of the experiments was 10 mm. Even though 5 mm is a recommended spacing in Australia, 10 mm to 12 mm has also been proposed for areas where homeowners are unlikely to wear high heel shoes and ease of litter cleaning is required, larger board spacing could be a worse-case scenario, and due to ignition source used (methenamine tablets as opposed to actual firebrands), ignition could not be achieved using 5 mm board spacing in this simple setup [26,27]. Two deck board thicknesses were used by Macindoe [35], 2 cm and 5 cm. Two cm is a more common board thickness and also corresponds to those used in this study as well (2.5 cm). Eight different wood species were used, namely (by lowest to highest wood density): radiata pine, cypress pine, mountain ash, merbau, jarrah, redgum, spotted gum, and grey ironbark. Under conditions of no external radiant heat (i.e., cone heater switched off and ignition achieved using methenamine tablets), no sustained flaming ignition was observed for 2 cm board thickness for MC (dry basis) 12–14%. As the MC was lowered, sustained FI was observed for some of the species at 2 cm board thickness. Once the board thickness was increased to 5 cm, all eight wood species sustained FI when the deck boards were oven dried. In general, it was noted that it was easier to ignite wood decking types with lower densities.

Hasburgh et al., [36] conducted preliminary experiments to determine wall ignition vulnerabilities exposed to burning decking assemblies. In their experiments, the decking assemblies were 609 mm by 711 mm, and these were attached to a wall fitted with heat flux gages and thermocouples. Two ignition sources were considered: a below-deck flame exposure using a propane burner, and an above-deck test using a Class A burning brand (largest), both taken from the SFM test method above. For the Class A firebrand ignition experiments, the experimental data showed that wind speeds of 2.9 m/s and 5.4 m/s had the highest temperature and heat flux on the wall from the burning decking assembly. Hasburgh et al. [36] suggested that test methods developed in their paper and the data obtained can be used to gain insight into how a burning wood deck contributes to structural ignition. However, as pointed out by the authors, a great deal of work remains to be done, as the number of experiments were limited [36].

Few research studies have investigated the vulnerabilities of fencing assemblies. In experiments at the NIST in Gaithersburg (MD, USA), fencing assemblies were ignited by a burner and flame spread was measured under wind conditions [37]. Since the NIST in the USA has no large-scale wind tunnel facilities for fire experimentation, the work was not performed in a wind tunnel, but rather outside, with wind being provided by a fan. It was reported that flame spread was affected by the wind direction with respect to fencing assemblies and mulch was needed for fires to propagate along fencing assemblies.

Ignition of landscape mulches are an obvious danger for firebrand shower exposures. While there have been some studies of mulch ignition in the literature [38,39], none of these studies have investigated the ignition of mulch installed in realistic building configurations exposed to wind-driven firebrand showers, conditions seen in real WUI fires. As a final comparison, Beyler et al. [39] is the only work to systematically investigate mulch ignition on realistic scales. The mulch beds were contained in 0.6 m square containers. In their work, shredded hardwood mulch was held at 13.3% MC. Different wood cribs, so called Class A, B, C, 0.5 C, and 0.25 C, were used to simulate firebrands. They observed it was possible to only produce FI in shredded hardwood mulch at 13.3% MC when using a Class A flaming firebrand. In their work, FI was defined as whether spread of visible flame front propagation after firebrand (crib) deposition was observed. For the reader not familiar with a Class A firebrand, this is a very large wood crib (30.48 cm 9 30.48 cm × 5.715 cm). None of the smaller sized firebrands used could produce flaming ignition. No wind speed was applied in these experiments, and in WUI fires, wind is known to be critical for WUI fire spread. Other than [26-31] no other prior literature studies, investigated showers of firebrands on realistic scale assemblies.

WUI fires require far more experimental data to be able to develop and validate the next generation fire models to tackle this global problem [5]. The overall dearth of experimental data and standard test methods on firebrand showers limits the ability to design the next generation of communities to resist WUI fires to these types of exposures. The objective of this paper is to summarize, compare, and reconsider the results from previous experiments [26-31]. Here, multiple experimental campaigns undertaken by the authors over several years were reconsidered to yield additional analysis in order to present new data that was not elucidated previously. The new data will provide insights to prevent home losses from firebrands in WUI fires.

## 2. Experimental Description

The overall series of experimental campaigns described in the paper have been presented elsewhere [26-31]. Therefore, the focus here is a brief description of experimental conditions (see Table 1) as well as the experimental apparatus (the full-scale continuous-feed firebrand generator (FS-CF-FG), a.k.a. the NIST continuous-feed Dragon) which used for all experiments to produce firebrand showers. The FS-CF-FG was made of two parts: a feeding part and a firebrand producing part (see Figure 2). Two hundred grams of wood pieces, firebrands before combustion, are stored in the container and are fed into the firebrand producing part every 15 s. Firebrands for all experiments were produced from Douglas-fir wood pieces with the dimensions of 7.9 mm × 7.9 mm × 12.5 mm. Firebrands produced from this size of Douglas-fir wood pieces were confirmed to be similar size range to firebrands produced from trees (wildland fires) and WUI fires [40,41].

The size and the mass of firebrands, collected by water pans, were measured via a separate set of experiments. The average projected area and the average mass of firebrands are 1.0 cm^2^ and 0.05 g with uncertainties of ±10% for 6 m/s 8 m/s. The difference of the average projected area or the mass of firebrands under 6 m/s and 8 m/s are negligible; both being within uncertainties.

As the wind plays an important role in WUI fire spread, all experiments were performed under wind. The unique wind tunnel facility located in Building Research Institute (BRI, Tsukuba, Japan) was used for experiments. The firebrand flux from the FS-CF-FG was 17.1 g/min for all cases. All experiments were performed under 6 m/s or 8 m/s wind, and not all the firebrands from the FS-CF-FG reached the experimental target. These wind speeds are selected as they are within the range of those observed in actual large outdoor fires such as the Angora fire in 2007 [40] or the Beppu-city fire in 2010 [42]. The temperature and humidity were recorded in each experiment. The difference of arrival firebrand flux at the target is discussed later.

### 2.1. Mulch Beds with Re-Entrant Corner Assembly

**Figure 1.**
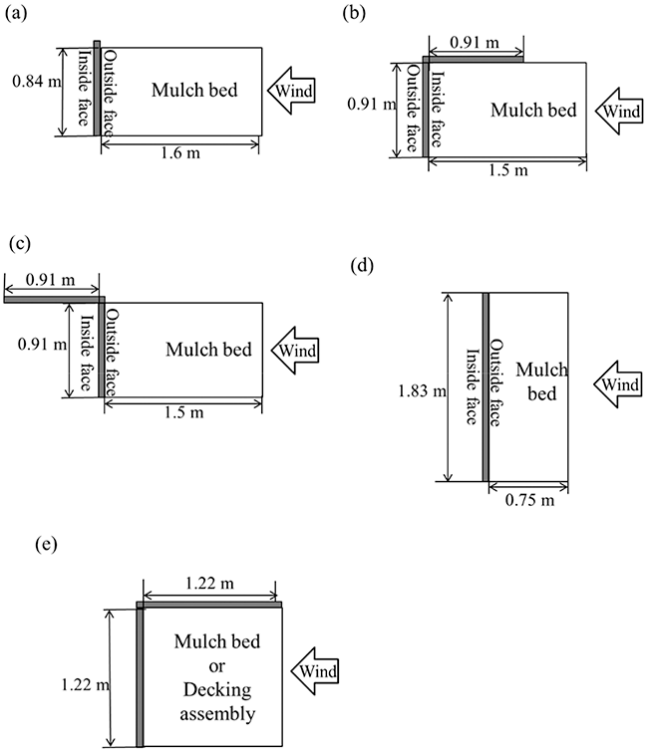
Mulch bed dimension for fencing assembly experiments (**a–d**) [29] and for corner assembly (**e**) [26-28,30,31].

**Figure 2.**
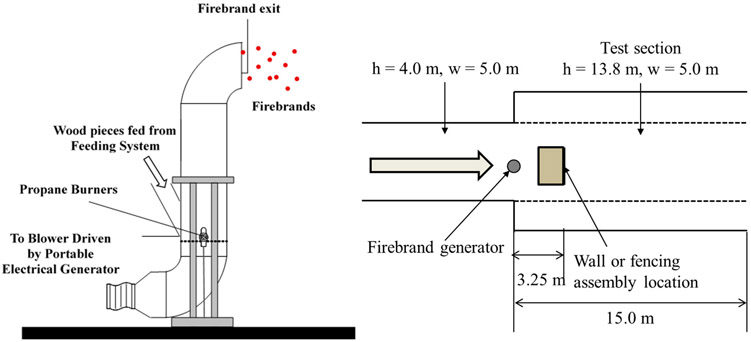
Image of experimental apparatus, the firebrand generator (the NIST Dragon).

A series of experiments were performed in order to investigate the vulnerability of various mulch types, such as shredded hardwood mulch, Japanese cypress wood chip mulch, and pine bark nugget mulch. The mulches were placed inside a re-entrant corner assembly (see Figure 1e) in order to simulate a realistic situation, as it would be in a home. The dimensions of the mulch beds were kept the same, 1.22 m × 1.22 m × 0.051 m, for all cases. Fuel Moisture Content (FMC) of shredded hardwood mulch was varied in order to see the FMC effect [30] while Japanese cypress wood chips and pine bark nuggets were oven-dried [31]. For some cases, the separation distance between the ground and the siding fitted to the wall assembly was varied (102 mm and 203 mm), and the effect to a surrounding re-entrant corner assembly from mulch ignitions was also investigated [31].

### 2.2. Fencing Assembly and Mulch Beds

Ignition of fencing assemblies by firebrand showers was also investigated. The same shredded hardwood mulch (discussed in Section 2.1) was placed in front of privacy fence assembly to investigate the vulnerability to firebrands. The configurations of fencing assemblies were varied in order to see the differences of firebrand showers on ignition (see Figure 1a-d). The shredded hardwood mulch was placed in front of (upwind side of) the fencing assemblies. In Figure 1b, the shredded hardwood mulch was placed inside the re-entrant corner fencing assembly. Some of the experiments were performed without shredded hardwood mulch in order to see the vulnerability to the fencing assemblies themselves. The mulch bed dimensions were varied to adjust to the respective fencing assembly, but the area was kept constant—1.44 m^2^—the same as experiments in Section 2.1.

### 2.3. Decking Assembly with Re-entrant Corner

Experiments were performed with three different decking boards, Douglas-fir, redwood, and cedar, which are common in the USA. None of the decking boards were treated with fire retardant. Decking assemblies were placed inside the re-entrant corner assembly, which is similar to the mulch bed location discussed in Section 2.1. The deck board gap was varied from 0 mm (no gap), 5 mm (normal practice), and 10 mm, for 8 m/s experimental cases. In the case of 10 mm gap under 8 m/s wind speed, only experiments with redwood was performed. Decking boards were facing the wind in parallel, as this configuration was suggested in a workshop prior to the experimental series as the most likely scenario in WUI communities [43].

## 3. Results and Discussion

**Figure 3.**
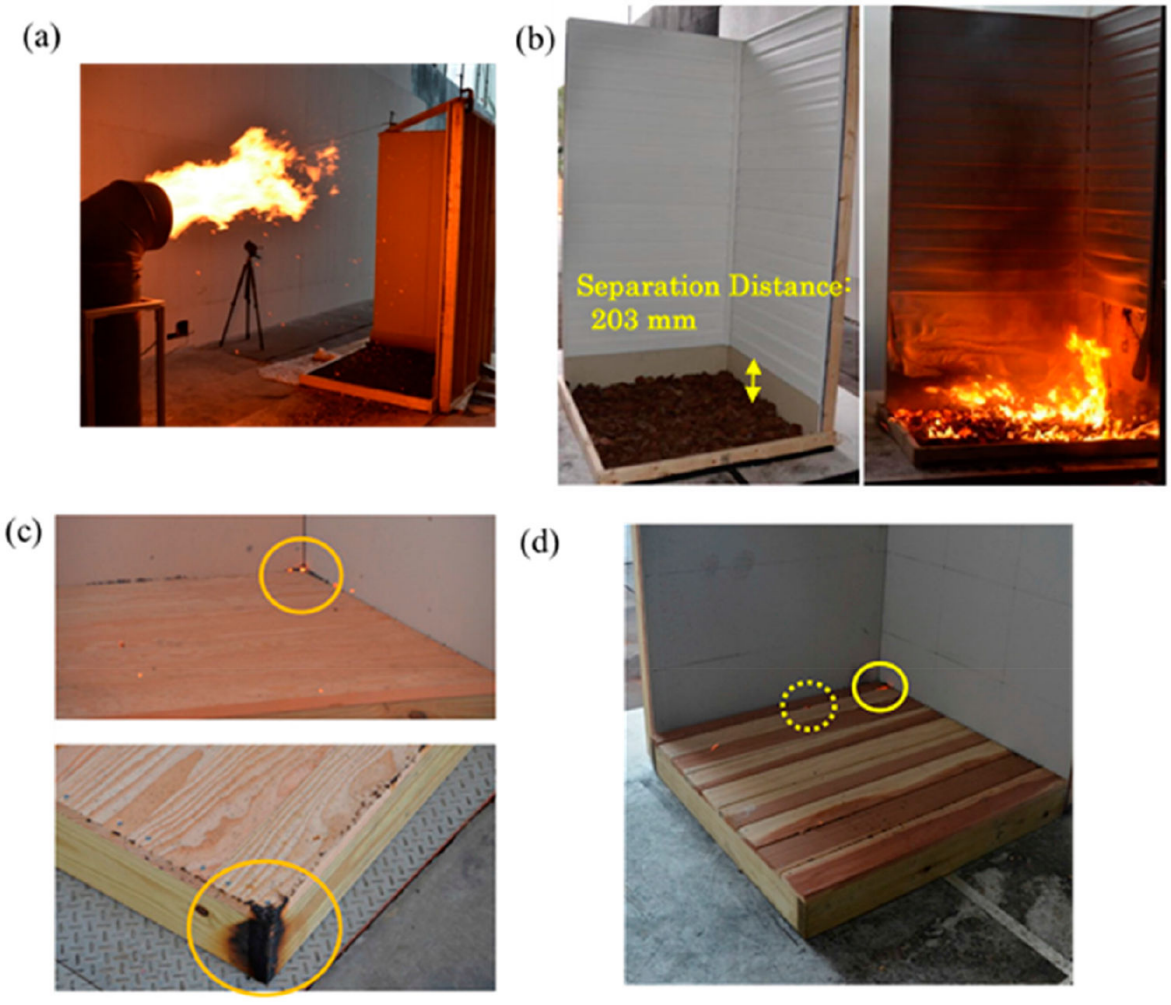
Images of experiments (**a**) shredded hardwood mulch with FMC 11% (6 m/s) [30] (**b**) Pine bark nugget mulch with 203 mm separation distance (6 m/s) [31] (**c**) Douglas-fir decking assembly with 0 mm gap spacing (8 m/s) [28] and (**d**) Redwood decking assembly with 5 mm gap spacing (8 m/s) [27].

Some of the experimental images are sampled and shown in Figure 3. Three ignition regimes were observed—no ignition at all (represented as NI), smoldering ignition but no flaming ignition (represented as SI), and smoldering ignition to flaming ignition (represented as FI). The time to ignition was measured, and results were compared. The experiments with shredded hardwood mulch beds were reported in [30,31], results were consolidated for this comparison as both data are similar and differences within uncertainties were considered. The temperature and humidity were recorded for each experiment and no effect on time to ignition was found. It was found FMC has larger effects on time to ignition. The firebrand flux landing on a fuel bed/decking assembly (shown in Figure 1e) is 7.36 and 6.18/m^2^ s for 6 and 8 m/s, respectively.

### 3.1. Comparison with Other Mulches, and Different FMC

**Figure 4.**
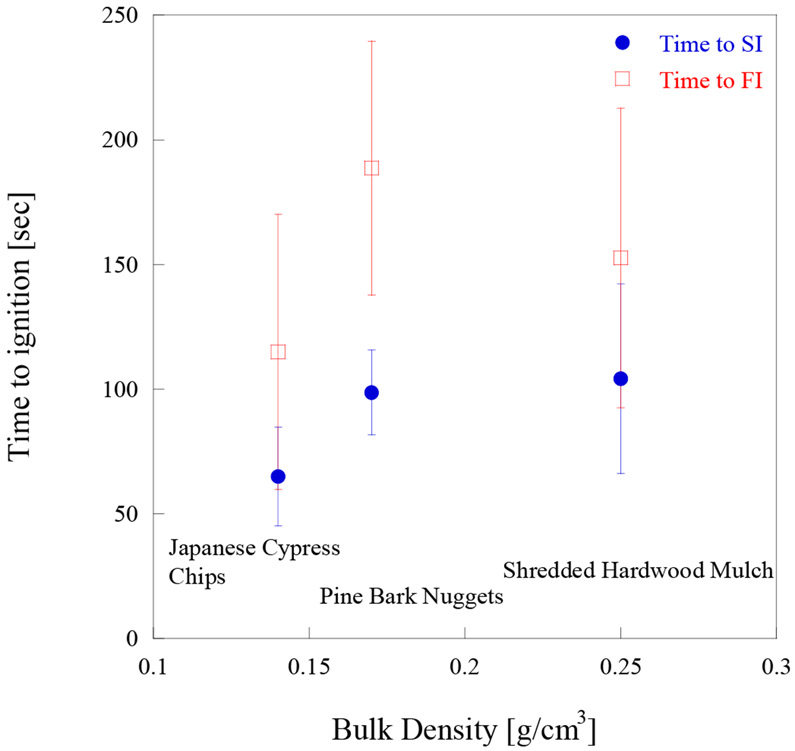
Time to ignition vs. the mulch bed bulk density on different mulch beds under 6 m/s wind.

Figure 4 shows the relationship between bulk density of each mulch utilized in the experiments and time to ignition (SI and FI), under a 6 m/s wind speed. It was observed that for pine bark nugget mulch it was more difficult to reach flaming ignition (FI) with firebrands than with shredded hardwood mulch or Japanese cypress chip mulch. The times to reach SI for pine bark nugget mulch and shredded hardwood mulch were similar, as shown in Figure 4. Shredded hardwood mulch is made from oak, Japanese cypress chip mulch is made from cypress, and pine bark nugget mulch is made from pine (bark). Of the three types of mulches used in these experiments, shredded hardwood mulch had the highest bulk density. It is well known that higher density wood boards are more difficult to ignite than lower density wood boards [44]. As thicker wood samples take longer to ignite [44], similar behavior was observed in the case of pine bark nugget mulch, as it contained the thickest individual pieces. The transition from SI to FI was more difficult for pine bark nugget mulch, as the pine bark nugget mulch beds naturally have large gaps among the individual pieces, and therefore the heat loss was large.

Flame spread to the re-entrant corner assembly under a 6 m/s wind speed were also investigated (Table 2). Here, the worst-case scenario was ignition in the presence of a 203 mm separation distance between the ground and the siding fitted to the wall assembly. Flames in both Japanese cypress chip mulch and pine bark nugget mulch reached the wall assembly beyond the 203 mm separation distance. Flames in shredded hardwood mulch could not reach the wall assembly when the separation distance was 203 mm. Shredded hardwood mulch was not only relatively hard to ignite by firebrands, but also the flame duration was short compared with other two mulches types used here.

**Figure 5.**
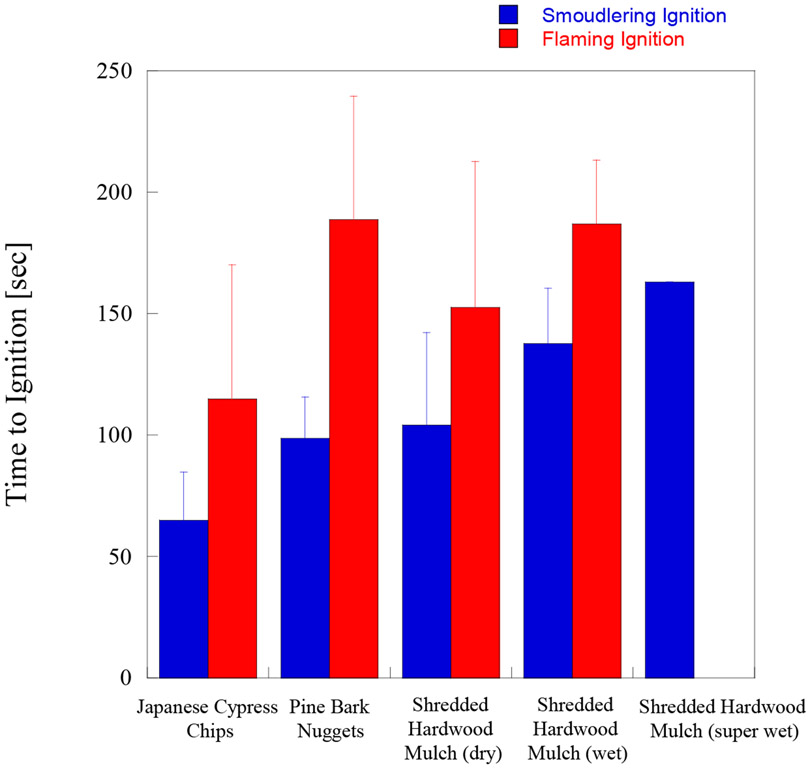
Comparison of time to ignition on the mulch bed of different mulch and FMC under 6 m/s wind.

Figure 5 shows the comparison of time to ignition under a 6 m/s wind. It is clear that time to SI for all dry mulch are shorter than the those for ‘wet (FMC 9% to 40%)’ or ‘super wet (FMC over 40%)’ shredded hardwood mulches. Time to reach FI for wet shredded hardwood mulch (FMC 9% to 40%) was similar to pine bark nugget mulch. Time for transition from SI to FI for wet shredded hardwood mulch (FMC 9% to 40%) was shorter than that for pine bark nugget mulch.

### 3.2. Decking Assembly Literature Studies

**Figure 6.**
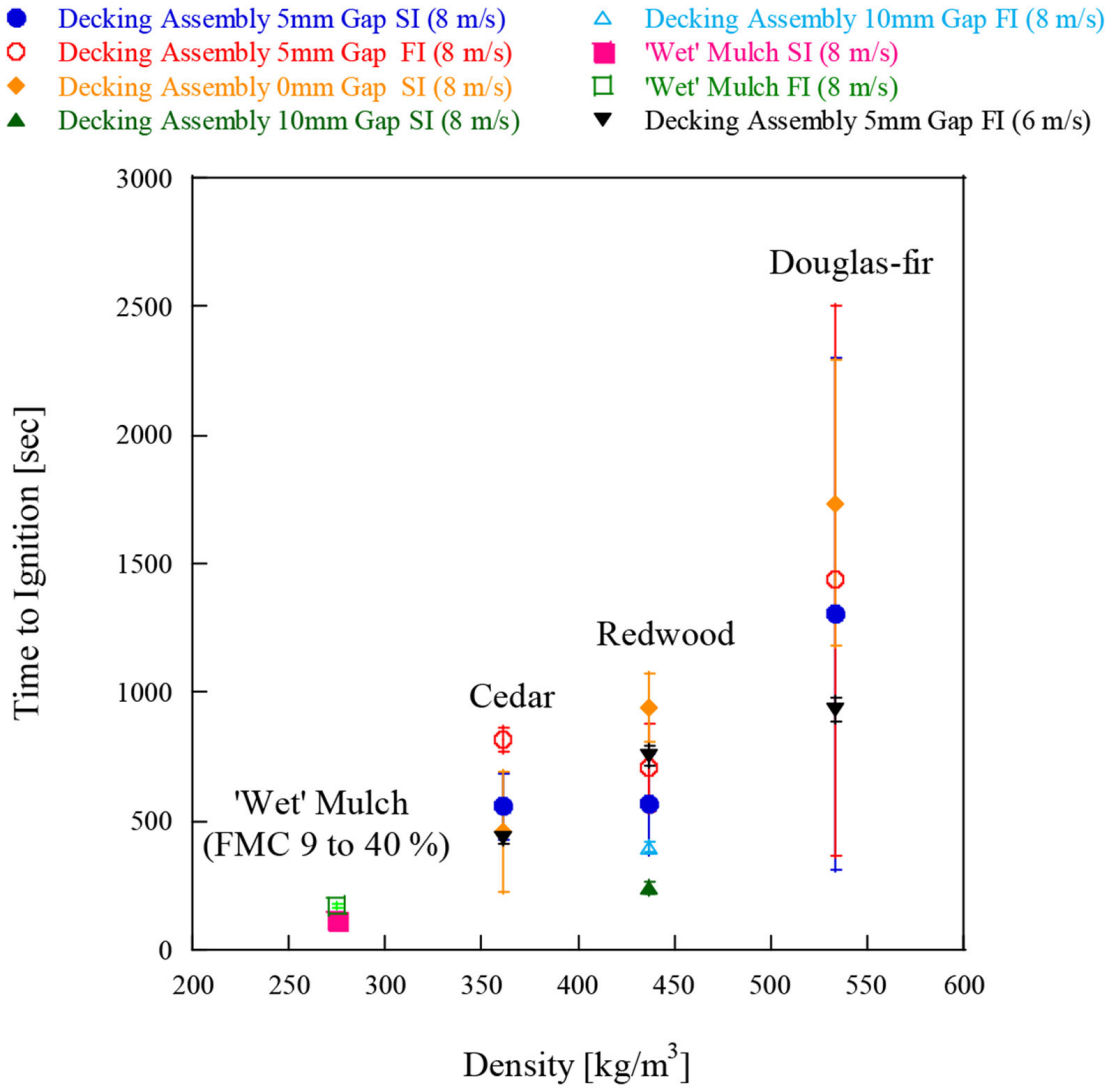
Time to ignition of decking assembly with various gap spacing under 8 m/s. The data for ‘wet’ mulch (FMC 9% to 40%) (8 m/s) and 5 mm gap decking assembly (6 m/s) were added for information. Please note that no data exist for 10 mm gap results for cedar and Douglas fir.

Wood decking assemblies are considered to be one of several vulnerabilities around houses to firebrand ignition and have been of interest to a few studies [26-28]. Different gap sizes showed different ignition vulnerabilities; larger gaps resulted in decking assemblies being more vulnerable to firebrands. Results were compared with shredded hardwood mulch with the same dimensions (1.22 m × 1.22 m) for simplicity, as shown in Figure 6.

**Figure 7.**
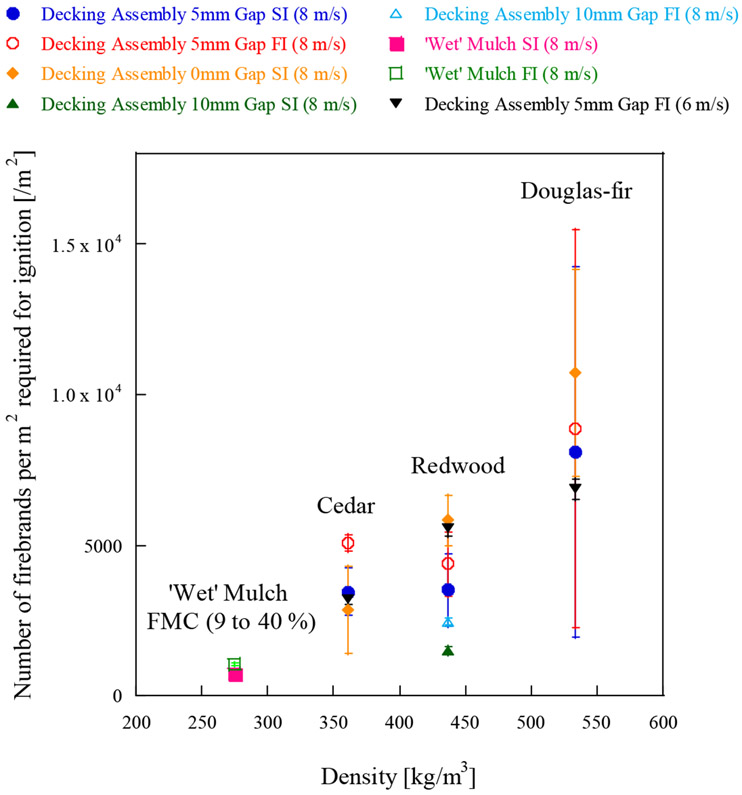
Number of firebrands per m^2^ for ignition of decking assembly with various gap spacing under 8 m/s. The data for ‘wet’ mulch (FMC 9% to 40%) (8 m/s) and 5 mm gap decking assembly (6 m/s) were added for information. Please note that no data exist for 10mm gap results for Cedar and Douglas-Fir.

All the experimental conditions are described in Table 1. As decking boards were not oven dried for this experimental series, and the MC varied between 9% to 15%, the comparison was made with ‘wet’ shredded mulch, whose FMC varies from 9% to 40%. Time to ignition (SI or FI) under 8 m/s are compared in Figure 6, first, then the number of firebrands per m^2^ for ignition are compared in Figure 7. Figures 6 and 7 contain the data from the decking assembly under a 6 m/s wind speed for reference [26]. As expected, the comparison reveals that the time to SI or FI for decking assemblies were at least twice as long than that for ‘wet’ shredded hardwood mulch (FMC 9% to 40%), the only exception is for 10 mm gap. The time to SI or FI for 10 mm gap decking assembly is similar to that for ‘wet’ hardwood mulch (FMC 9% to 40%). Time to ignition under 6 and 8 m/s wind conditions for 5 mm gap decking assembly is similar (Figure 6), while past studies [27,28] showed the actual mass required to ignite a 5 mm gap decking assembly under 8 m/s were much smaller than the mass required under 6 m/s. Figure 7 shows the time to ignition would be almost the same if the difference of firebrand flux under two wind speeds is taken into consideration. This shows if the firebrand flux is similar for 6 and 8 m/s wind, the time to ignition is similar despite the fact the firebrand accumulation location or ignition points may be different.

### 3.3. Effect of Mulch Bed Dimension and Firebrand Flux on Ignition Vulnerabilities

In the past, shredded hardwood mulch was used as a fuel bed to examine the ignition vulnerability of mulch itself [30] or in combination with other structural assemblies around houses [29]. Those investigations revealed that dry shredded hardwood mulch is vulnerable to firebrand showers, being ignited by firebrands within only a few minutes. The question remains—is there any particular fuel bed dimension which is safer than others in WUI communities? Past research was reanalyzed to answer this question. Here, the aspect ratio, defined as $\frac{\text{the length (parallel to the wind) of fuel beds}}{\text{the width (perpendicular to the wind) of fuel beds}}$ was considered. The thickness of the fuel beds was kept the same, 51 mm. Details of experimental conditions are shown in Table 1. All experiments in this comparison were performed under an 8 m/s wind. Under these conditions, the firebrand landing flux (the number of firebrands landing on the mulch bed per m^2^ per second) varied from 5.6 to 9.7, with aspect ratio changing from 0.53 to 2.4. A clear dependence of firebrand flux on the aspect ratio was not observed.

**Figure 8.**
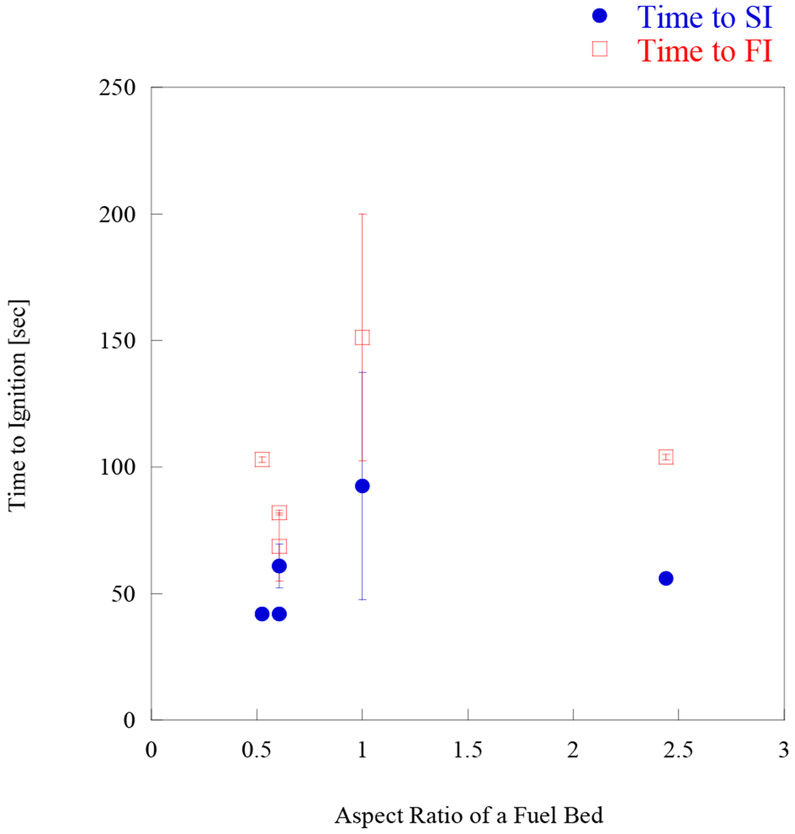
Time to ignition vs. aspect ratio of a fuel bed under 8 m/s wind.

**Figure 9.**
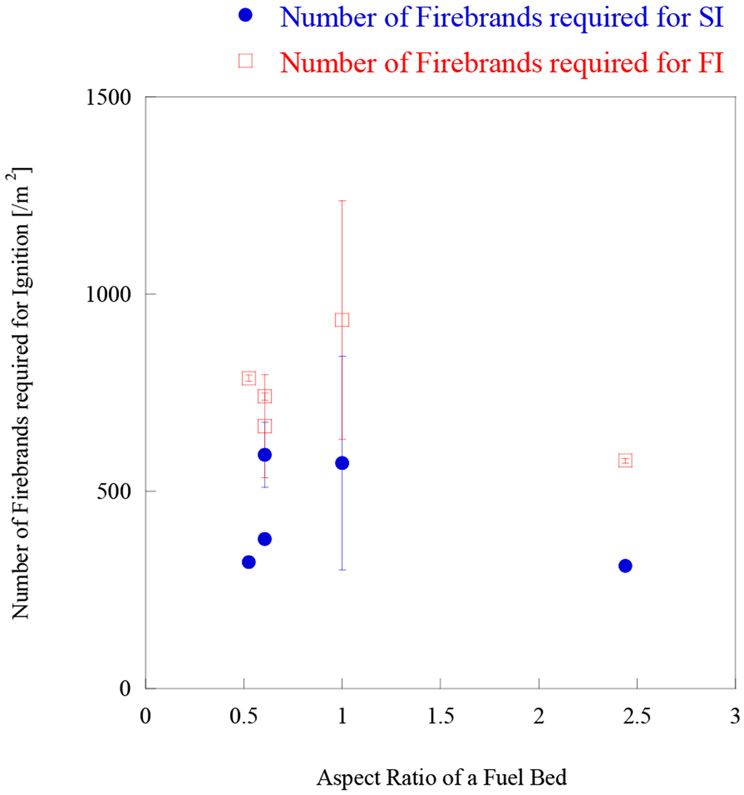
Number of firebrands required for ignition vs. aspect ratio of a fuel bed under 8 m/s wind.

Time to ignition as well as the number of firebrands required for ignition per unit area (m^2^), for both SI and FI, is shown in Figures 8 and 9, respectively.

As all dimensions have a different firebrand landing flux, the number of firebrands for ignition per unit area (m^2^) is also presented. Figure 8 shows that at the aspect ratio 1 (the corner assembly), the time to ignition has a peak for both SI and FI, and the time to ignition are relatively constant in other aspect ratios. Aspect ratio 1 has larger uncertainties due to the number of repeated experiments, and the assembly is different from the other cases. The fencing assembly does have small gaps between the boards while the corner assembly does not. This peak is considered to be caused by assembly differences rather than fuel bed aspect ratio. More investigation is necessary in the future. Figure 9 considered the difference of firebrand flux, in this case, the peak at aspect ratio is much less than time to ignition for both SI and FI. With the firebrand landing flux considered, the difference among fencing assemblies are smaller.

It is well known that the transition from SI to FI is a complicated phenomenon; in this case, while the applied wind was the same for all experiments, the actual wind speed around the assembly may differ as the assembly configuration is not the same. As noted above, the fencing assembly has certain gaps between fencing boards, while re-entrant corner assembly does not have such gaps. This would certainly influence the SI-to-FI transition. As mentioned in [29,30], FI in all experiments spread to the assemblies, which resulted in the fencing/corner assembly combustion.

**Figure 10.**
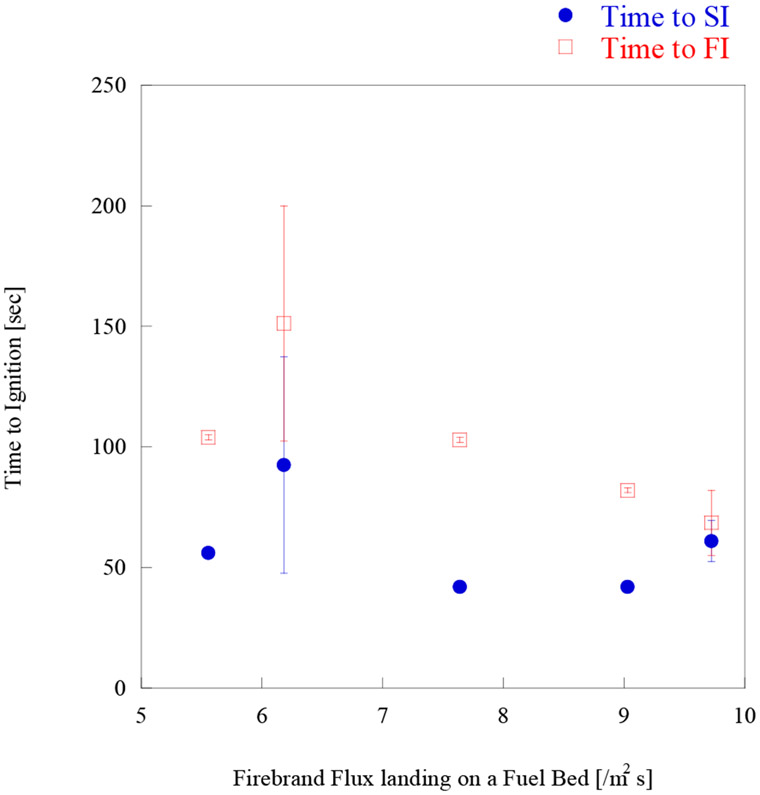
Time to ignition vs. firebrand landing flux under 8 m/s.

The comparison between firebrand flux and time to ignition are also made in Figure 10. Results suggest that time to ignition on mulch was not sensitive to firebrand landing flux between 5/m^2^ s to 10/m^2^ s, considering the uncertainties of experimental data. Bench-scale experiments have shown that as the firebrand flux goes down, the time to ignition increased and eventually firebrands cannot ignite samples [45]. This means that despite the difference of firebrand landing flux on mulch bed, the shortest time to SI on shredded hardwood mulch may be around 1 min. The minimum firebrand flux on shredded hardwood mulch still needs to be investigated.

### 3.4. Practical Implications of this Study

There is clearly a need to have globally accepted test methods to carry out laboratory experiments to understand firebrand ignition of various components found in homes. Globally agreed test methods will enable a collective understanding of how to best harden communities from firebrand exposures. These unique experimental datasets will serve as a starting point for these discussions.

## 4. Summary

Firebrands are known to be one of the driving factors of fire propagation in WUI fires. An attempt was made to summarize the ignition vulnerabilities of various combustibles often seen around houses to firebrand showers. Not surprisingly, all the mulch types considered were vulnerable to ignition from firebrand showers. For the cases where the mulch bed FMC was varied, ignition was still observed for high FMC, suggesting the overall dangers of mulch beds near homes. The bulk density and the size of the individual mulch pieces influence the time to ignition. A higher mulch bulk density requires more time to reach SI. Larger individual mulch piece size (and space among these pieces) requires more time for SI-to-FI transition due to the heat loss. Perhaps the most interesting result is that redwood decking assemblies with 10 mm gap between boards may be ignited within similar times to mulch beds, under certain conditions (wet mulch FMC of 9% to 40%). In particular, it was shown that wood decking assemblies may be ignited within 15 min when exposed to firebrand showers used in the studies. Within the range of firebrand flux tested, the time to ignition on mulch was insensitive to the firebrand flux when considering experimental uncertainties.

Additional future work is needed to help link the range of firebrand fluxes used in these studies to actual firebrand fluxes measured from actual WUI fires. Yet regardless of the actual WUI fire firebrand flux data, homeowners in WUI fire prone areas should strongly consider not using wood mulch or wood decking assemblies, as these were easily ignited in these laboratory scale studies. It is important to grasp that more datasets are needed for firebrand processes to begin to tackle this growing global problem.

The experimental studies in this paper have focused on housing construction common in the USA, yet wood decking assemblies are becoming common in Japan. Recent studies discuss the WUI fire situation in Europe [46], but to the authors knowledge, such detailed experimental studies of homes with features common to Europe have not been undertaken. With the development of the firebrand generator technology described in this paper, it is desired such studies will follow in Europe.

There is also the need to have globally accepted test methods necessary to harden communities to WUI fire exposures as well as globally accepted definitions related to the WUI fire problem. WUI includes various stages of urban developments and forest/wildland, which makes it difficult to have globally accepted test methods [47]. Such work is currently underway in ISO TC92 (Fire Safety), as part of ISO TC92/WG14 Large Outdoor Fires and the Built Environment Working Group [48].

## Figures and Tables

**Table 1.** Summary of experimental conditions.

Combustible Element Investigated	Parameter		Other Information	Wind Speed [m/s]	Reference
Shredded hardwood mulch	FMC	oven-dry, wet (9–40%) and super wet (over 40%)	Mulch dimension (see Figure 1e)	6, 8	[30]
Shredded hardwood mulch, Japanese cypress chip mulch, Pine bark nugget mulch	Separation Distance between mulch surface to sidings	102 mm and 203 mm	Mulch was oven-dry, mulch dimension (see Figure 1e)	8	[31]
Fencing assembly	Fence/mulch configuration	See Figure 1a-d	Mulch was oven-dry FMC of fencing assembly was approximately 10%	8	[29]
Decking assembly	Decking materials	Douglas-fir, Redwood, Cedar	Decking dimension (see Figure 1e) Gap 5 mm FMC of decking assemblies was around 10% (6 m/s) and 12% (8 m/s)	6, 8	[26,27]
Decking assembly	Gap between deck boards Decking materials	Douglas-fir (0 mm), Redwood (0 mm and 10 mm), Cedar (0 mm)	Decking dimension (see Figure 1e) FMC of decking assemblies was from 10% to 16%	8	[28]

**Table 2.** Transition success of mulch ignition to wall assembly (SD: separation distance) [31].

	Shredded Hardwood Mulch	Japanese Cypress Chip Mulch	Pine Bark Nugget Mulch
6 m/s	102 mm SD	203 mm SD	203 mm SD

## Data Availability

The data is this study is available from the authors upon request.
